# Variability in age and size at maturation, reproductive longevity, and long-term growth dynamics for Kemp's ridley sea turtles in the Gulf of Mexico

**DOI:** 10.1371/journal.pone.0173999

**Published:** 2017-03-23

**Authors:** Larisa Avens, Lisa R. Goshe, Lewis Coggins, Donna J. Shaver, Ben Higgins, Andre M. Landry, Rhonda Bailey

**Affiliations:** 1 National Marine Fisheries Service, Southeast Fisheries Science Center, NOAA Beaufort Laboratory, Beaufort, North Carolina, United States of America; 2 US Fish and Wildlife Service, Yukon Delta National Wildlife Refuge, Bethel, Alaska, United States of America; 3 National Park Service, Padre Island National Seashore, Corpus Christi, Texas, United States of America; 4 National Marine Fisheries Service, Southeast Fisheries Science Center, Galveston Laboratory, Galveston, Texas, United States of America; 5 Texas A&M University at Galveston, Galveston, Texas, United States of America; 6 Florida Fish and Wildlife Conservation Commission, Fish and Wildlife Research Institute, St. Petersburg, Florida, United States of America; Florida State University, UNITED STATES

## Abstract

Effective management of protected sea turtle populations requires knowledge not only of mean values for demographic and life-history parameters, but also temporal and spatial trends, variability, and underlying causes. For endangered Kemp’s ridley sea turtles (*Lepidochelys kempii*), the need for baseline information of this type has been emphasized during attempts to understand causes underlying the recent truncation in the recovery trajectory for nesting females. To provide insight into variability in age and size at sexual maturation (ASM and SSM) and long-term growth patterns likely to influence population trends, we conducted skeletochronological analysis of humerus bones from 333 Kemp’s ridleys stranded throughout the Gulf of Mexico (GOM) from 1993 to 2010. Ranges of possible ASMs (6.8 to 21.8 yr) and SSMs (53.3 to 68.3 cm straightline carapace length (SCL)) estimated using the “rapprochement” skeletal growth mark associated with maturation were broad, supporting incorporation of a maturation schedule in Kemp’s ridley population models. Mean ASMs estimated from rapprochement and by fitting logistic, generalized additive mixed, and von Bertalanffy growth models to age and growth data ranged from 11 to 13 yr; confidence intervals for the logistic model predicted maturation of 95% of the population between 11.9 and 14.8 yr. Early juvenile somatic growth rates in the GOM were greater than those previously reported for the Atlantic, indicating potential for differences in maturation trajectories between regions. Finally, long-term, significant decreases in somatic growth response were found for both juveniles and adults, which could influence recruitment to the reproductive population and observed nesting population trends.

## Introduction

The conservation history of the endangered Kemp’s ridley sea turtle (*Lepidochelys kempii*) has been one of extremes. Although historically individual Kemp’s ridleys were occasionally reported from the Gulf of Mexico (GOM) and western North Atlantic, their origins were obscure and the location of the constituent breeding areas remained an enigma for decades [1, 2]. Then in 1961, film footage obtained in 1947 by Andres Herrera came to light, showing thousands of female Kemp’s ridleys nesting simultaneously at a single location in Rancho Nuevo, Tamaulipas, Mexico, highlighting the extremely limited spatial scope of reproductive habitat for the species [2, 3]. However, by the time this information was revealed, the nesting population had dwindled to several thousand and, by the 1980s, only a few hundred nesting females remained, presumably due to a combination of poaching and fishery bycatch [4]. Following extensive, bi-national conservation efforts by the USA and Mexico, the number of nesting females began to increase exponentially, offering encouragement for population recovery and the possibility that the species might even be downlisted under the US Endangered Species Act within the foreseeable future [5, 6]. Then in 2010, coincident with the massive *Deepwater Horizon* (DWH) oil spill in the northern GOM [7], nest numbers suddenly decreased by more than 30% relative to 2009 levels [4]. While nest numbers subsequently rebounded to some extent, exponential growth has not resumed and a further decrease in 2014 raised concerns regarding future recovery prospects for the species [4]. Recent estimates indicate that the nesting population remains reduced by 90% relative to historic levels [8].

The DWH oil spill had immediate, deleterious impacts on juvenile Kemp’s ridleys and habitat in the affected area [9] that may influence population dynamics for these turtles in the long term [5]. However, it is unlikely that the spill was a principal, underlying cause for the 2010 decrease in nesting; at the time the spill occurred on April 20, turtles that were reproductively active during that year would likely have already migrated to Rancho Nuevo and not been in the vicinity of the spill to be directly affected [4]. Given this disconnect, other factors have been proposed for the change in nesting population trajectory beginning in 2010 that might influence population components independently, or in combination to varying degrees. One is that a large-scale mortality event involving large juvenile and adult females might have occurred following the 2009 nesting season, and it has been suggested that changes in the prevalence of these life stages in strandings data be evaluated [10]. However, population models have indicated that the decline may be due to longer-term causes, such as a chronic decrease in survival or recruitment to the reproductive population [4]. Another possibility is that, as the population increased exponentially, density-dependent factors relating to reproductive and/or foraging habitat might have begun to decrease egg survival [4] and/or influence the frequency with which females nest within and among years, possibly limiting the total size of the population [11]. Finally, related to these density-dependent effects, changes in somatic growth and resulting age at maturation could also be influencing recruitment to the reproductive population [4].

Investigating all of these possibilities requires long-term, baseline life-history, demographic, and vital-rate data, which are difficult to obtain for sea turtles due to their variable somatic growth, lack of external age-related characteristics, delayed maturation, and highly migratory behavior [12]. With respect to age and growth, it is known that the Kemp’s ridley is the smallest sea turtle species at maturation and exhibits relatively rapid somatic growth overall, relative to other hard-shelled sea turtles [13]. In addition, observations made of captive-reared [14] and head-started female Kemp’s ridleys that later returned to nest [15] indicate that wide ranges of age and size at sexual maturation, or ASM and SSM, are possible. Although these data offer valuable insights, a number of questions remain, such as: How variable are ASM and SSM for wild populations? How long do Kemp’s ridleys live after reaching reproductive maturity and continue to contribute to the population, i.e., what is the reproductive longevity? Does the species exhibit sex- and region-specific differences in size-at-age relationships and growth rates [13]? Finally, are there long-term somatic growth patterns for Kemp’s ridleys that might influence recruitment to the reproductive population and be reflected in the recently-observed population trends?

One approach for collecting comprehensive age and growth data over long time scales is skeletochronology, which is the analysis of growth marks deposited on a cyclic basis in skeletal elements [12]. The primary bone used for skeletochronology in hard-shelled sea turtles is the humerus, as it retains the greatest number of skeletal growth marks used for age and growth studies [16]. When appropriately validated, each growth mark within the humerus can be linked with a calendar year, an age estimate, and somatic measure (i.e., carapace length). Furthermore, taking the difference between successive carapace length estimates can provide an annual somatic growth rate for each increment, providing a window back in time for individuals and, collectively, populations [12, 17]. In the current study, we apply skeletochronology to humeri collected from Kemp’s ridleys stranded throughout the GOM over two decades prior to 2010, to provide detailed age and growth data for the species in this region. The results of this study offer insights into variability in life-history characteristics among individuals and long-term growth patterns that are likely to influence population trends.

## Materials and methods

### Sample collection and processing

Humeri were obtained through cooperation with the national Sea Turtle Stranding and Salvage Network operating throughout the GOM, to maximize geographic representation while recognizing that stranding location does not necessarily reflect foraging location. Network participants collected front flippers from Kemp’s ridley sea turtles that either stranded dead, or were alive but debilitated and were subsequently euthanized. Stranding location and date were reported, along with carapace length, which was typically measured as straightline carapace length, notch to tip (SCL), although at times only curved carapace length, notch to tip (CCL) measurement was collected. CCL was converted to SCL using the following equation, based on 218 paired measurements for Kemp’s ridleys ranging from hatchlings to adults:

$$\text{SCL}=0.9566\left(\text{CCL}\right)-0.2105\;\left({\text{R}}^{2}=0.996\right)$$

For a portion of these turtles, sex was either characterized through examination of the gonads during necropsy (n = 165), or inferred from tail length for turtles larger than minimum adult size as reported by Caillouet et al. [15] (n = 7).

Humeri from 333 Kemp’s ridleys stranded dead in the GOM between 1993 and 2010 just prior to the DWH oil spill were selected for analysis and processed; samples originated from Texas (60%), Louisiana (6%), Mississippi (2%), Alabama (2%), and the Gulf coast of Florida (30%). SCL ranged from 4.2 to 69.1 cm (mean = 45.7±14 SD), with 27.3% of the samples collected from females, 24.3% from males and 48.4% from turtles of unknown sex. Percentages of turtles in the sample from the different 10 cm SCL size bins are as follows: 4.2–9.9 (2.1%), 10–19.9 (1.5%), 20–29.9 (7.8%), 30–39.9 (26.7%), 40–49.9 (18.0%), 50–59.9 (24.6%), 60–69.9 (19.2%).

Humeri were removed from the flippers, cutting away as much surrounding tissue as possible, after which they were boiled, dried in the sun for a minimum of two weeks, and then processed according to the methods described by Avens and Snover [12] and Avens et al. [18]. Although the left humerus was preferred for consistency, the right was processed instead when the left was not available. Briefly, a 2–3 mm thick cross-section was taken of the diaphyseal shaft just distal to the muscle insertion scar near the delto-pectoral crest, which was then decalcified using Cal-Ex II fixative/decalcifier (Fisher Scientific). A freezing-stage microtome (Leica) was then used to collect 25 μm thick sections, which were stained using Ehrlich’s hematoxylin. Stained sections were mounted on microscope slides in 100% glycerin and sequential, partial images of each section were taken at ×4 magnification using a compound microscope and transmitted light (Olympus BX41) in conjunction with image capture software (Olympus Microsuite and cellSens). Partial images for each humerus were combined using Adobe Photoshop to yield a calibrated, composite digital image of the entire cross-section that could be used for analysis. Two readers (L. Avens, L. R. Goshe) independently examined each image to determine the number and placement of the lines of arrested growth (LAGs) that define the outer edges of individual skeletal growth marks and worked together to reach consensus if a discrepancy arose. Once consensus was reached, diameter was measured for each LAG in a section, as well as the total humerus section diameter (HSD).

### Age estimation

Application of skeletochronology is based on the assumption that skeletal growth marks are deposited in a predictable, cyclic pattern [12]. Previous analyses of humeri from known-age Kemp’s ridleys, as well as characterization of the seasonal nature of bone deposition for these turtles along the Atlantic coast, have supported annual LAG deposition (i.e., 1 LAG/yr) [19]. To provide additional validation, we analyzed humeri of two known-age turtles that had stranded dead and were included in our sample; both had been tagged using coded wire tags (CWT, Northwest Marine Technologies). One was tagged and released as a hatchling at the nesting beach at Rancho Nuevo, Tamaulipas, Mexico, and the other was captive-reared at the National Marine Fisheries Service Laboratory in Galveston, Texas, USA, and released as a yearling. After ages were estimated using skeletochronological analysis, known ages were provided to the readers for comparison.

Skeletochronological age estimation in sea turtles can be impeded by a phenomenon called resorption, which is the modification and/or destruction of early LAGs due to expansion of the porous bone at the core during growth [16]. To account for any LAGs that might be resorbed in the humeri in our sample, we developed a stepwise correction factor based on the relationship between LAG number and diameter (e.g., [17, 18]) starting with the diffuse first-year LAG termed the “annulus” [19]. As resorption cores tend to be amorphous with inconsistent boundaries, the diameter of the innermost LAG that could be measured was used as a proxy for resorption core diameter, which was then substituted for LAG diameter in the correction factor to estimate the number of lost LAGs [17, 18]. For those bones where the annulus was observed, age was determined directly from LAG counts. Because annual LAGs are deposited at the outer edge of the bone during the late winter/early spring [19] and peak hatching occurs during the summer, deposition of the annulus actually denotes ~0.75 years of age, the next LAG 1.75 yr, and so on. As a result, each LAG was assigned an age *x*.75 yr, instead of a whole year. In cases where LAGs were predicted to have been resorbed, the estimated number of LAGs within the resorption core was added to the number of observed LAGs to yield a final age estimate. Age estimates at stranding were adjusted to the nearest 0.25 yr based on stranding date and mean hatch date for the population [13].

### SCL back-calculation and somatic growth

The ability to predict body size from skeletal growth mark measures is contingent upon the strength of the relationship between these two variables. Previous analyses have demonstrated a strong correlation between medial width (MW; the diameter of the humerus at the sectioning site), as well as HSD and LAG diameters and SCL for loggerhead (*Caretta caretta*) and green sea turtles (*Chelonia mydas*) [17, 18, 20–22] and between MW and SCL for Kemp’s ridleys [19]. Here, we characterized the relationship between HSD and SCL for Kemp’s ridleys, incorporating minimum values measured for hatchlings of 1.66 mm and 3.74 cm, respectively, as a starting point. We also used samples from five turtles captured, tagged, measured, and released that spent sufficient time at large to exhibit somatic growth prior to stranding, to validate the relationship between LAG diameter and SCL for the species (e.g., [17, 18, 20]). Briefly, assuming that one LAG was deposited per year, each LAG was assigned a calendar year to determine which LAG would have been deposited closest to the time of tagging. The HSD:SCL relationship in combination with the body proportional hypothesis (BPH [20]) was applied to estimate SCL from LAG diameter and this estimate was then compared to the SCL measured at tagging. In addition, to account for rapid somatic growth between LAG deposition in early spring and later capture during the summer foraging season, SCL estimates adjusted according to available somatic growth data (*see Supplemental information*) were also compared with SCL measured at tagging.

Following characterization and validation of the HSD:SCL relationship, SCL was estimated for every measurable LAG in each humerus section and annual somatic growth rates were calculated by taking the difference between successive SCL estimates [17, 18]. Generalized additive mixed models (GAMMs [23]) that could accommodate repeated sampling from each turtle were used to evaluate the potential influence of different covariates such as sex, age, SCL, and year on growth response, with turtle ID incorporated as a random, individual-specific effect [17, 18]. As ovarian development in wild Kemp’s ridleys has been reported to begin around 50 cm SCL [15], separate GAMMs were also run for data from turtles smaller and larger than that size. Furthermore, because a high degree of co-concurvity was observed between age and SCL (0.74 and 0.96, respectively, on a scale of 0 to 1 with 1 representing the worst-case scenario), as in previous sea turtle studies [17, 18, 22], it was necessary to separate these covariates into different models. All GAMMs incorporated an identity link and robust quasi-likelihood error function and were implemented using the *mgcv* and *nlme* packages in the statistical program R version 3.2.2 [23, 24]). Significance of model factors was evaluated using *t* ratio statistical inference for nonparametric covariates (sex) and nonparametric *F* ratio test for continuous covariates (age, SCL, year). During stepwise model runs, those factors not significant at the level of p ≤ 0.05 were discarded in subsequent model runs, for which best-fitting models were then characterized as those with the lowest Akaike’s information criterion (AIC) values in conjunction with the highest adjusted *R*^2^ values.

To look for regional differences in growth patterns, growth rates back-calculated for the GOM in the current study were compared to those available for the Atlantic. The most extensive data for the Atlantic were from years prior to 2000 [13], yielded by skeletochronological analysis of 144 humeri (n = 178 back-calculated growth increments) from Kemp’s ridley sea turtles ranging from 14 to 61 cm SCL that stranded along the coasts of North Carolina, Virginia, and Maryland. As a result, for this aspect of the study, the GOM data were restricted correspondingly and size class-specific (i.e., stratified into bins each encompassing 10 cm SCL) means and standard errors for the two regions for this time period were compared. In addition, Faben’s modified von Bertalanffy growth curve was fit to the Atlantic growth data [13] and GOM data (*see following section for description of methods*), to allow comparison of curve fits.

### Maturation attribute estimation

Attaining reproductive maturation is associated with a marked decrease in somatic growth in reptiles and amphibians, which corresponds with an abrupt decrease in LAG spacing toward the outer edges of bones, an attribute termed “rapprochement” [25–27]. We observed a significant decrease in LAG spacing toward the lateral edges of humerus sections from some of the larger (>50 cm SCL) Kemp’s ridleys in our sample, consistent with rapprochement (comparison of pre- and post-rapprochement growth, p < 0.001, Mann-Whitney Rank Sum Test). As a result, the LAG at this transition was designated as the rapprochement LAG signifying maturation [17]. Age and size at maturation (ASM and SSM) were inferred from the age and SCL estimates associated with the rapprochement LAG. Reproductive longevity was estimated by counting the number of LAGs between the onset of rapprochement and the outer edge of the bone, under the assumption that LAGs continue to be deposited annually in reproductively mature individuals [17].

ASM and SSM were also evaluated using logistic models to generate cumulative probability distributions of reaching maturation at a given age or SCL (i.e., maturation ogives) (e.g., [28]). Finally, ASM was estimated using the SCL-at-age data (both back-calculated for each LAG and final at stranding) by applying two different approaches, to evaluate correspondence between predictions. The first method involved fitting a smoothing spline using a GAMM that could account for random, individual effects potentially introduced by longitudinal sampling [29]. Smoothing splines and 95% confidence intervals were fit for males and females separately to assess potential differences and then for the total population [17]. For the second approach, we repeatedly sampled the back-calculated somatic growth rate data to extract a single data point for each turtle in the sample and these nonparametric bootstrap samples were then used to estimate the parameters *k* and *L*_*∞*_ for Fabens modification of the von Bertalanffy growth curve [30, 31]. Randomized re-sampling of the growth-rate data was conducted 1,000 times to describe uncertainty in the von Bertalanffy parameters [17]. For both the smoothing spline and von Bertalanffy growth curve, ASM was characterized as the age associated with different SSM estimates from the published literature, as well as those predicted from rapprochement and the maturation ogive in the current study.

## Results

### Validation analyses

Age estimated for the first known-age Kemp’s ridley that was tagged as a hatchling and stranded at 37.8 cm SCL was 3.75 yr and this turtle’s actual age was 3.68 yr. The second known-age Kemp’s ridley that was tagged after being reared in captivity for approximately 1 yr and then released was also estimated to be 3.75 yr. Its SCL at stranding was 50.8 cm SCL, considerably larger than the first known-age turtle’s, but its actual age was also 3.75 yr.

The relationship between SCL and HSD was best characterized as allometric, as found in other sea turtle studies [17, 18, 20, 22], with slope (*b*) = 3.26 and the allometric proportionality coefficient (*c*) = 0.93. This relationship was applied together with the body proportional hypothesis (BPH; [20]) to estimate SCL from the diameter of the LAG deposited closest to the time of tagging for the five tagged turtles in the sample. No significant difference was found between measured SCL and SCL estimated through back-calculation without further adjustment (p = 0.60, paired T-test; S1 Table) and mean difference was 1.2 cm SCL. However, because LAGs are deposited in the late winter/early spring [19], with growth external to the LAG observed as early as April, and Kemp’s ridleys can exhibit rapid somatic growth during the summer/early fall foraging season, estimated SCL was also adjusted using available growth rate information (see Supporting information—Appendix). The mean discrepancy between adjusted, estimated SCL and measured SCL was 0.4 cm and the difference was not significant (p = 1.0, Wilcoxon Signed Rank Test; S1 Table).

### Age estimation

Of the total sample, 12 small turtles 4.2 to 20.0 cm SCL (mean = 10.9 cm SCL ± 6.2 SD) exhibited either no LAGs or the beginning of an annulus and were therefore assigned ages <1 yr to the nearest quarter year, according to stranding date relative to mean hatching date [13]. Mean age for these turtles was 0.4 yr ± 0.3 SD. Of the remaining humeri in the sample, 147 retained the diffuse first-year LAG, or annulus, either fully or in part and the turtles from which these humeri were collected ranged from 20.3 to 57.8 cm SCL (mean = 36.2 cm SCL ± 7.5 SD). For those annuli that were measurable, back-calculated SCL at the time of annulus deposition ranged from 15.8 to 24.5 cm SCL (mean = 20.4 cm SCL ± 2.0 SD; n = 137).

A stepwise correction factor based on the relationship between LAG number and LAG diameter was developed to estimate the number of LAGs missing in those humeri exhibiting resorption of the first-year mark. The first step was to describe this relationship for the 147 humeri retaining all or some portion of the annulus, to generate the first order correction factor (CF1). It was found that a second order polynomial (equation) provided the best fit to these data (330 LAG number:LAG diameter pairs):

$$\text{CF1}:\text{ LAG diameter}=-0.2159{\left(\text{LAG number}\right)}^{2}+3.4551\left(\text{LAG number}\right)+4.4999\;\left(R^{2}=0.73\right)$$

For the remaining 174 humeri in the sample, resorption core diameters for 125 were smaller than the largest LAG diameters described by CF1; these humeri originated from stranded turtles 34.6 to 64.4 cm SCL (mean = 53.3 cm SCL ± 7.1 SD). Resorption core diameter for these 125 samples was substituted for LAG diameter in CF1 to estimate the number of LAGs lost to resorption. Based on the estimated number of lost LAGs, each observable LAG in these 125 humeri was assigned a number to develop a second order correction factor (CF2), comprising 980 LAG number:LAG diameter data pairs. A fifth order polynomial (equation) provided the best fit for this relationship:

$$\text{CF2: LAG diameter}=0.0004\;{\left(\text{LAG number}\right)}^{4}-0.0124{\left(\text{LAG number}\right)}^{3}+0.0242{\left(\text{LAG number}\right)}^{2}+2.2421\left(\text{LAG number}\right)+5.533\;\left(R^{2}=0.91\right)$$

CF2 was applied to estimate the number of LAGs lost in resorption cores for the remaining 49 humeri in the sample, which was added to the number of observed LAGs to yield an age estimate for each turtle. Turtles in this final group ranged from 54.7 to 69.1 cm SCL (mean = 54.7 cm SCL ± 3.2 SD). Age estimates for the entire sample at stranding ranged from 0 to 30.25 yr (mean = 6.8 yr ± 5.7 SD).

### ASM, SSM, and reproductive longevity

A total of 55 turtles in the sample of male, female, and unknown sex exhibited LAG compaction consistent with rapprochement; at stranding, sizes for these turtles ranged from 54.5 to 69.1 cm SCL (mean = 62.5 ± 3.0 SD) and ages at stranding were estimated at 9.0 to 30.25 yr (mean = 16.8 ± 4.6 SD). With regard to maturation attributes inferred from rapprochement LAGs for this sub-set of the sample, no significant difference was found between sex-specific SSMs (p = 0.07, Mann-Whitney Rank Sum Test), with an overall mean of 61.3 cm SCL and range from 53.3 to 68.3 cm SCL (Table 1). No significant difference was found between female and male ASM (p = 0.22; Mann-Whitney Rank Sum Test), with estimates ranging from 6.8 to 21.8 yr (mean = 12.2 ± 3.5 SD). Similarly, no sex-specific differences in reproductive longevity were found (p = 0.96, t-test) and individuals were estimated to live up to 10 yr after reaching maturation (mean = 4.5 ± 2.2 SD).

**Table 1:** Summary of attributes related to reproductive maturation for Gulf of Mexico Kemp’s ridley sea turtles (*Lepidochelys kempii*), as estimated from the Line of Arrested Growth (LAG) associated with the onset of rapprochement.

*Attributes related to reproductive maturation*							
SSM (cm; SCL notch-to-tip)	*n*	Mean	SD	Min	Max	Median	CV
All	*55*	61.3	3.3	53.3	68.3	61.8	0.05
Females	*26*	62.0	4.0	53.3	68.3	62.0	0.06
Headstarted females^a^	*49*	61.8	1.8	58.1	65.8	-	0.03
Captive females^b^	*14*	52.6	-	44.8	58.2	-	0.07
Males	*24*	60.3	2.5	56.1	65.1	60.7	0.04
ASM (yr)							
All	*55*	12.2	3.5	6.8	21.8	11.8	0.29
Females	*26*	12.9	4.3	6.8	21.8	11.8	0.34
Headstarted females^a^	*49*	15.3	3.6	9.7	22.8	-	0.24
Captive females^b^	*14*	8.1	2.0	5.0	12.0	-	0.25
Males	*24*	11.3	2.5	7.8	18.8	10.8	0.22
Adult stage duration (yr)							
All	*55*	4.5	2.2	1	10	5	0.50
Females	*26*	4.6	2.5	1	10	5	0.55
Males	*24*	4.5	2.0	2	8	5	0.43

Attributes include age and size at maturation (ASM and SSM) and reproductive longevity. Values reported in the published literature for head-started and captive Kemp’s ridleys are provided in the shaded rows, for comparison.

^a^[15];

^b^[14].

**Fig 1:**
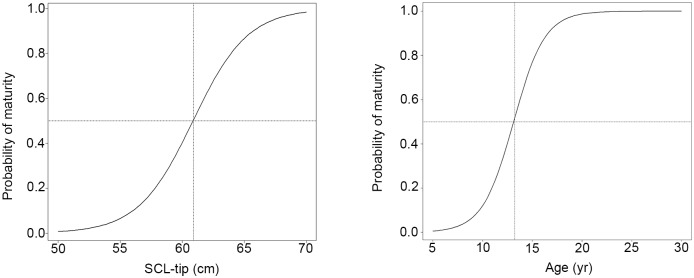
Logistic curves fit to bootstrapped, back-calculated Gulf of Mexico Kemp’s ridley (*Lepidochelys kempii*) size and age data. A. straightline carapace lengths (notch-tip; SCL) and B. age estimates. Model fits predict that 50% of the population would become mature by 61.0 cm SCL and age 13.3 yr.

**Fig 2:**
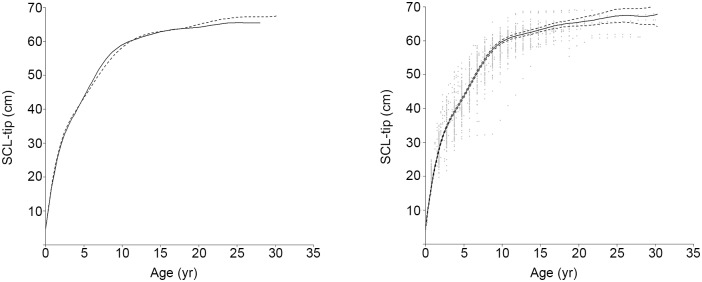
Generalized Additive Mixed Model (GAMM) smoothing splines fit to all Gulf of Mexico Kemp’s ridley (*Lepidochelys kempii*) estimates of age and back-calculated Straightline Carapace Length (notch-tip; SCL). A. Sex-specific spline fits for females (dashed line, n = 546 datapoints from 91 individuals, r^2^ = 0.94, p < 0.001) and males (solid line, n = 514 datapoints from 82 individuals, r^2^ = 0.94, p < 0.001); B. SCL-at-age data, spline fit (solid line), and 95% confidence interval (dashed lines) for all data from females, males, and turtles of unknown sex (n = 1,729, r^2^ = 0.94, p < 0.001).

Assigning an age and back-calculated SCL estimate to each LAG yielded 1,729 age:SCL data pairs. Logistic models fit to these bootstrapped data estimated mean SSM at 61.0 cm SCL (95% CI 59.9 to 62.2 cm SCL) and mean ASM at 13.3 yr (95% CI 11.9 to 14.8 yr) (Fig 1). GAMM splines fit to sex-specific SCL-at-age data indicated no differences between males and females (Fig 2A) and, as a result, a spline was fit to all SCL-at-age data (Fig 2B). The overall spline fit was significant (p < 0.001; adjusted R^2^ = 0.94) and random, individual effects were significant as well (p < 0.0001, log-likelihood ratio test). For the minimum SSM predicted from rapprochement (53.3 cm SCL), the spline predicted a minimum ASM of 7.5 yr (Table 2). Mean SSMs predicted from the logistic model fit (61.0 cm SCL) and rapprochement (62.0 cm SCL) corresponded with ASM estimates of 11 and 13 yr; however, the total range of mean ASM estimates corresponding with mean SSMs reported in the literature ranged from 10 to 18.5 yr (Table 2).

**Table 2:** Summary of estimates of Kemp’s ridley (*Lepidochelys kempii*) Straight and Curved Carapace Lengths (SCL and CCL, cm) associated with Size at Sexual Maturation (SSM) and associated Age at Sexual Maturation (ASM) reported in the literature (gray-shaded rows).

Source for SCL at maturation (SSM)	SCL	CCL*	Previous ASM estimates	Rapprochement LAG ASM	Mean spline age	Mean ogive age	Mean bootstrap VB age	*k*	Linf
*Females*									
GOM—Current Study Min	53.3	55.9	-	6.8	7.5		6.5	0.25	65.9
Headstarted Min^a^	58.1	61.0	9.7^a^	-	9		8.4	0.25	65.9
GOM Mean^b^	60	62.9	10^b^	-	10		9.5	0.25	65.9
Atlantic Mean^c^	60	62.9	10–17^c^	-	10		9.5	0.25	65.9
Headstarted Mean^a^	61.8	64.8	15.3^a^	-	12		11	0.25	65.9
GOM—Current Study Mean	62	65.0	-	12.9	13		11.2	0.25	65.9
GOM Mean^d^	64	67.1	10–11^d^	-	16		14	0.25	65.9
Atlantic Mean^d^	64	67.1	12–13^d^	-	16		14	0.25	65.9
GOM/Atlantic Mean^e^	65	68.2	15.7^e^	-	18.5		17.1	0.25	65.9
*Males*									
GOM US Current Study Min	56.1	58.9	-	7.8	8.5		7.5	0.25	65.9
GOM Current Study Mean	60.3	63.3	-	11.3	10.5		9.7	0.25	65.9
*All*									
GOM Current Study Ogive Mean SSM	61	64.0	-			13.3 (95%CI 11.9 to 14.8)			

SSM estimates from the current study either associated with the rapprochement line of arrested growth (LAG) or yielded by the maturation ogive are interspersed in the un-shaded cells. ASM estimates were generated from rapprochement LAG analysis, fitting a spline using a generalized additive mixed modeling (GAMM) approach, fitting a maturation ogive, and fitting Faben’s modified von Bertalanffy growth curve.

^a^[15];

^b^[32];

^c^[13];

^d^[78];

^e^[50]

*SCL converted to CCL using formula: CCL = (SCL + 0.2105)/0.9566.

### Growth

Because two consecutive, measurable LAGs were needed to calculate a growth rate, sample size for back-calculated growth increments (n = 1,263) was less than that for SCL-at-age. Each increment was associated with a calendar year (1988 to 2009) and age (corresponding with the initial LAG for the increment), SCL (mean back-calculated SCL for the increment), and sex (male, female, unknown). Bootstrapping these data to fit Faben’s modified von Bertalanffy growth curve yielded an intrinsic growth rate (*k*) of 0.25 and asymptotic SCL (*L*_*∞*_) of 65.9 cm SCL. ASM estimates associated with minimum and mean SSMs predicted from the current study and reported from previous studies corresponded with minimum ASM estimates of 6.5 to 8.4 yr and mean ASM estimates ranging from 9.5 to 17.1 yr (Table 2).

**Fig 3:**
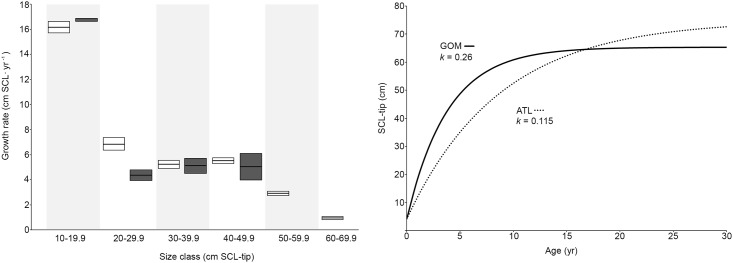
Comparison of somatic growth for Kemp’s ridleys (*Lepidochelys kempii*) in the Gulf of Mexico (GOM; white; n = 535) and the Atlantic US coast (ATL; dark gray [13]; n = 178) prior to the year 2000. A. 10 cm size class-specific back-calculated annual somatic growth rates. Horizontal black lines represent means and bars extending to either side represent 1 SE; B. Faben’s modified von Bertalanffy growth curves where *k* is the von Bertalanffy growth coefficient. See Table 3 for additional growth curve parameters.

Comparison of growth data back-calculated for Kemp’s ridleys stranded in the GOM (n = 535) vs. the Atlantic [13] prior to 2000, the time frame for which data from both regions were available, indicated that overall rates were similar, with the exception that growth was much faster in the GOM for turtles in the 20 cm SCL size class (Table 3; Fig 3A). Fitting Faben’s modified von Bertalanffy growth curve to bootstrapped pre-2000 GOM data yielded *k* = 0.26 and *L*_*∞*_ = 65.3, contrasting with Atlantic values of *k* = 0.115 and *L*_*∞*_ = 74.9 cm SCL for the same time period and emphasizing divergence in early growth rates (Fig 3B).

**Table 3:** Annual somatic growth rate data for 10 cm Straightline Carapace Length (SCL) size classes for Kemp’s ridley sea turtles (*Lepidochelys kempii*) in the Gulf of Mexico (GOM) and along the US Atlantic coast yielded by skeletochronological analysis and capture-mark-recapture studies.

Mean SCL (cm)	Growth rates (cm/yr)				
	Skeletochronology			Mark-Recapture	
	Current study GOM (1998–2009)	Current study GOM (pre-2000; *n* = 535)	Atlantic [13] (pre-2000; *n* = 191)	GOM [79]	GOM [80]
10–19.9	15.9 ± 2.6 SD	16.1 ± 2.4 SD; 0.3 SE			
	(2.9 to 20.6)	(6.4 to 20.6)			
	*n* = 103	*n* = 58			
20–29.9	6.8 ± 3.3 SD	6.9 ± 3.2 SD; 0.4 SE	4.4 ± 0.3 SE	-	3.42 ± 2.64 SD
	(0.0 to 15.4)	(0.8 to 15.4)			(0.0 to 8.26)
	*n* = 131	*n* = 52			*n* = 12
30–39.9	5.7 ± 3.3 SD	5.2 ± 3.2 SD; 0.3 SE	5.1 ± 0.5 SE	4.6 ± 2.8 SD	5.5 ± 2.98 SD
	(0.0 to 13.1)	(0.0 to 13.1)		(1.2 to 9.4)	(1.25 to 8.92)
	*n* = 206	*n* = 97		*n* = 7	*n* = 8
40–49.9	5.5± 2.5 SD	5.5 ± 2.1 SD; 0.2 SE	5.0 ± 1.0 SE	6.2 ± 3.7 SD	3.3
	(0.0 to 12.1)	(1.3 to 11.7)		(2.9 to 13.0)	*na*
	*n* = 199	*n* = 87		*n* = 13	*n* = 1
50–59.9	2.6 ± 1.7 SD	2.9 ± 1.5 SD; 0.1 SE	-	4.6 ± 2.5	-
	(0.0 to 8.1)	(0.0 to 6.8)		(2.2 to 7.9)	
	*n* = 339	*n* = 150		*n* = 4	
60–69.9	0.6 ± 0.8 SD	0.9 ± 1.0 SD; 0.1 SE	-	-	-
	(0.0 to 4.6)	(0.0 to 4.6)			
	*n* = 285	*n* = 91			
**Von Bertalanffy growth curve parameters**					
	Time to maturation (SSM 60 cm SCL)	9.4 yr	10 to 17 yr		
	Growth coefficient *k*	0.26	0.115		
	Asymptotic length (L∞)	65.3 cm SCL	74.9 cm SCL		

**Fig 4:**
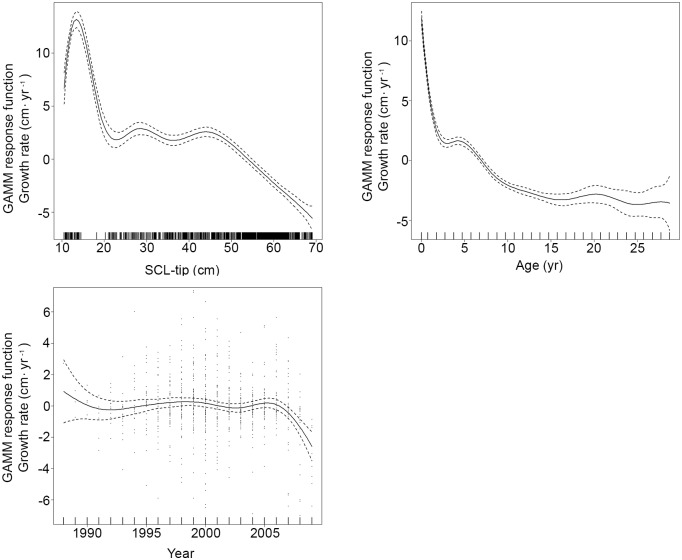
Summary of Generalized Additive Mixed Model (GAMM) graphical output for models incorporating all back-calculated somatic growth data for known-sex Gulf of Mexico Kemp’s ridley sea turtles (*Lepidochelys kempii*), Straightline Carapace Length (notch-tip; SCL) or age (yr), and calendar year (Year). Plots for those covariates exhibiting a significant influence on growth response are shown. Solid lines represent mean growth response centered around 0 and dashed lines represent the extent of the 95% Bayesian credible interval. The short, vertical lines above the horizontal axis (i.e., ‘rugs’) represent the distribution of samples for a given covariate. See S2 Table for sample sizes and statistical output.

**Fig 5:**
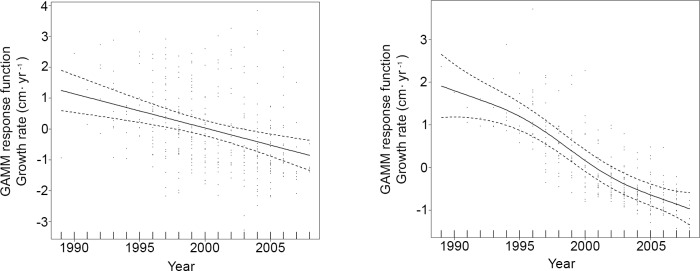
Generalized Additive Mixed Model (GAMM) spline fits (solid line) and 95% confidence intervals (dashed lines) for growth response relative to calendar year for back-calculated annual somatic growth data for Gulf of Mexico Kemp’s ridley sea turtles (*Lepidochelys kempii*) >50 cm Straightline Carapace Length (SCL). A. 50–59.9 cm SCL (n = 339). B. 60–69.9 cm SCL (n = 285).

Initial base models run were GAMM_SCL+Year+Sex_ and GAMM_Age+Year+Sex_ and incorporated all covariates and back-calculated growth increments. However, unknown sex was found to have a significant influence because these turtles disproportionately comprised small juveniles exhibiting rapid growth rates and it was necessary to restrict further analyses to known-sex individuals (n = 828 back-calculated annual growth increments). During these subsequent model runs, no sex-specific differences were found and GAMM_SCL_ and GAMM_Age+Year_ were found to have the best fit, with all three covariates exhibiting a significant influence on growth response (S2 Table; Fig 4A, 4B and 4C). Similarly, for juvenile turtles <50 cm SCL (n = 303), the best-fitting models included SCL and year and age and year (S3 Table). In general, juvenile growth response decreased with increasing size and age; however, short-term increases in growth were observed, particularly around ages of 2 and 5 yr and between 25 and 30 cm SCL. Although broad confidence intervals early in the time period represented by the growth data make trends difficult to interpret, growth response increased from 1995 to 2003 and then decreased through 2009 (S1 Fig). For large juvenile and adult Kemp’s ridleys >50 cm SCL (n = 525), the best-fitting models again incorporated SCL and year and age and year (S4 Table). Growth decreased with increasing SCL and age and, in contrast to the juveniles, also declined throughout the study period, from 1990 to 2009 (S2 Fig). As near-zero growth rates in adults can confound trend detection (e.g., [17]), separate GAMM splines were also fit to the 50 and 60 cm SCL size classes, with each showing a significant, consistent decrease in growth response over almost two decades (Fig 5).

## Discussion

Despite an extended period of increasing Kemp’s ridley sea turtle nesting trends from 1990 to 2009 that suggested recovery of this endangered species might be a near-term possibility, recent fluctuations and overall decreases in nest numbers 2010 to present have raised concerns regarding population status and future trends. As a result, in the most recent status review for the species, the US National Marine Fisheries Service (NMFS) elevated the recovery priority number for Kemp’s ridleys to the highest level, defining the species as one:

“…whose extinction is almost certain in the immediate future because of a rapid population decline or habitat destruction, whose limiting factors and threats are well understood and the needed management actions are known and have a high probability of success, and is a species that is in conflict with construction or other developmental projects or other forms of economic activity”

[4].

The Kemp’s ridley’s relatively restricted geographic distribution, small population size, and focused monitoring of nesting females, nests, and hatchlings as part of intensive conservation efforts has provided greater knowledge of some population characteristics relative to other sea turtle species [11]. Nevertheless, like other sea turtles, Kemp’s ridleys are highly migratory, fairly slow to mature, and exhibit no external age-related characteristics; therefore, detailed information regarding somatic growth rates and maturation attributes for wild turtles remains a significant data gap. Characterization of somatic growth rates and age distribution at first nesting has been highlighted as a management priority [4], as this information is needed to improve population modeling efforts that can increase understanding of recent changes in population trajectory and facilitate management and recovery [32]. The present study combines different analytical approaches based on skeletochronological age and growth data to provide comprehensive insight into size-at-age relationships, maturation attributes, and long-term somatic growth patterns for Kemp’s ridleys inhabiting the GOM from 1988 to 2010.

### Validation analyses

For those turtles with tagging histories, no significant difference was found between measured and back-calculated carapace lengths at tagging, similar to validation conducted for loggerhead [17,18,20] and green sea turtles [21, 22, 33]. This approach is particularly useful, as it confirms the predictable relationship between bone and somatic measures and provides the premise for back-calculating annual somatic growth rates. Because the LAG deposited closest to the time of tagging is identified assuming that one LAG is deposited each year, this correspondence provides additional, indirect support for the assumption of annual LAG deposition.

Size at deposition of the diffuse LAG, or annulus, representing approximately 0.75 yr of growth during the first year of life was estimated to be between 15.8 and 24.5 cm SCL, with a mean of 20.4 cm SCL. This size is comparable to the 21 cm SCL estimated at annulus deposition for Kemp’s ridleys stranded along the US Atlantic coast [13]. Witherington et al. [34] found juvenile Kemp’s ridleys 17.5 to 27.6 cm SCL (mean = 23.3 cm) associated with pelagic *Sargassum* in the eastern GOM and estimated them to be 1 to 2 years old. Similarly, Putman and Mansfield [35] captured and tracked pelagic juvenile Kemp’s ridleys 14.1 to 29.9 cm SCL (mean = 20.6 cm) in the same region. Putman et al. [36] modeled the time for water particles to travel from the primary Kemp’s ridley nesting beach to the northeast GOM as 1.2 yr, corresponding with 23.3 cm SCL, further corroborating early age and size estimates yielded by skeletochronological analysis in the current study.

### Size, age, and maturation

Due to the inability to follow individual sea turtles during the extensive habitat shifts and migrations undertaken throughout their lives, age at sexual maturation (ASM) has typically been inferred by fitting growth curves to somatic growth rates measured during life stage-specific mark-recapture studies and estimating age at a particular size at sexual maturation (SSM) (reviewed by [12]). As adult females become accessible for study when they emerge onto nesting beaches, while adult males remain elusive in the marine environment, the SSM used to infer ASM has often been assumed to be either the minimum or mean carapace length of nesting females for a given population [12]. Even characterization of minimum adult female size can be problematic, particularly for species such as Kemp’s ridleys where many hundreds of individuals may nest simultaneously, hindering identification of all neophytes [10]. Although assumption of single ASMs and SSMs offers some insight into possible maturation attributes, variability should be expected, as these characteristics represent the end result of individual growth trajectories influenced by multiple biotic and abiotic factors and trade-offs between growth, survival, and maturation [37, 38].

Correspondingly, recent studies have demonstrated considerable variability in both SSM and ASM in different sea turtle populations. Both green [39] and Kemp’s ridley [14] sea turtles reared in captivity their entire lives in the same environment displayed divergence in maturation attributes, with differences in SSM among individual green turtles spanning 32 cm (~30% of total size for adult green turtles); [39] and Kemp’s ridley ASM estimates differing by as much as 7 yr [14]. Head-started Kemp’s ridleys released into the wild and later observed at presumed first nesting exhibited similar variability, with SSM differing by 8 cm among individuals (~13% of adult turtle size) and the range of ASMs spanning 13 yr [15]. It might be expected that these differences would be reduced as a result of homogeneous captive conditions during part or all of the turtles’ lives. However, recent skeletochronological analysis of ASM and SSM for wild loggerhead sea turtles in the western North Atlantic revealed variability in maturation attributes comparable to at least some captive values [17], highlighting the potential importance of individual-specific genetic and phenotypic influences on growth and maturation [14].

Rapprochement analyses in the current study provided insight into possible minimum and mean SSMs for wild Kemp’s ridley sea turtles, as well as variability surrounding this life-history trait. Physiological initiation of maturation may start well prior to attaining size at which reproductive activity is initiated; ovarian development has been found to occur in wild female Kemp’s ridleys as small as 50 cm SCL, with the expectation that full maturation would not occur for at least several years following [40]. In the current study, minimum female SSM based on rapprochement was estimated to be 53.3 cm, consistent with this assumption, and similar to the 55 cm SCL mean minimum SSM observed for neophyte nesters [41]. While SSM as small as 44.8 cm SCL has been observed for Kemp’s ridleys in captivity [14], this is likely to reflect the unnatural environment experienced by the turtles under those conditions [41]. Conversely, minimum SSM for head-started females [15] was larger by 13.7 cm SCL compared to captive females [14] and 4.8 cm SCL greater compared to wild female Kemp’s ridleys in the current study. This discrepancy may reflect the influence of early captive rearing under optimal conditions for the head-starts, resulting in a “silver spoon effect” whereby circumstances early in life affect later growth trajectories [42]. With respect to mean female SSMs, our estimates of 62.0 cm SCL from rapprochement and 61.0 cm from the maturation ogive fall toward the lower end of the range of mean sizes previously used to estimate ASM (Table 2). Based on results of rapprochement analyses, mean female SSM was slightly larger than mean male SSM, perhaps reflecting selection for larger SSMs to maximize female reproductive output at maturation [43].

Minimum and mean female ASMs (6.8 yr and 12.9 yr, respectively) estimated from rapprochement LAGs were higher than those observed for captive females, but lower than those reported for head-started females (Table 1). We present here the first ASM values for male Kemp’s ridleys, which were not significantly different from those estimated for females. Mean rapprochement ASM was comparable to estimates yielded from size-at-age and growth models in the current study incorporating mean rapprochement SSM (12.9 and 11.2 yr, respectively), as well as the mean logistic model ASM (13.3 yr) (Table 2). Although the mean ASM estimates yielded by the current study fall within the central part of the range previously proposed for wild female Kemp’s ridleys (Table 2), the current results highlight the extent of variability for this trait (CV = 0.34; Table 1), similar to recently-reported observations from captive [14] and head-started [15] females. Together, these findings support the recommendation to incorporate a maturation schedule in Kemp’s ridley population models, as opposed to assuming knife-edge maturation at a particular age [10]. While a recent population model for the species incorporated gradual maturation between ages 9 and 14 yr [4], data yielded by the current study indicate that 95% of the population might mature between 11.9 and 14.8 yr. However, as ASM (along with SSM) should be expected to vary over time in response to different population pressures [41], these maturation attributes should be periodically characterized.

The ASM and reproductive longevity data presented herein along with those yielded by comparable analyses for western North Atlantic loggerheads [17] offer insight into different life-history approaches for these sea turtle populations. The Kemp’s ridley is the smallest sea turtle species, with estimates presented herein for minimum SSM of 53.3 cm SCL and mean SSM of 61.0 to 62.0 cm SCL for wild individuals. In contrast, for loggerheads inhabiting the same region, minimum and mean SSMs for females were estimated to be 75 and 90.5 cm SCL, respectively. While additional samples from large adult Kemp’s ridleys are needed to comprehensively describe maximum age for the species, no turtle in the current sample had an estimated age older than 30.25 yr; however, maximum ages estimated for loggerheads using this same analytical approach ranged from 70 to 77 yr [17]. Differences also manifested in reproductive longevity estimates, as Kemp’s ridleys were predicted to survive ≤10 yr after maturing (mean = 4.5 yr), which corresponds with re-sightings of tagged nesting females at Rancho Nuevo over time spans up to 9 yr [41]. However, this time frame is relatively short compared to loggerheads for which reproductive longevity may span decades (range 4 to 46 yr, mean = 19 yr) [17]. The discrepancy in reproductive longevity may be reflective of differences in survival between the two species, with a lower survival rate for adult Kemp’s ridleys in their foraging grounds, migratory corridors, or inter-nesting habitats. There are also differences in remigration intervals and the number of clutches of eggs laid by females from the two species; available data indicate loggerheads in the western North Atlantic nest on average every 3 to 4 yr, with a mean of 3 to 4 clutches per yr [44], while Kemp’s ridleys nest on average every 2 yr and lay a mean of approximately 2 clutches per yr [45]. However, there is evidence that the Kemp’s ridley re-migration interval increased from 2008 to 2016 and was approximately 3.5 years in the time period 2014 to 2016 [46]. Changes in remigration intervals due to decreased foraging resources, density-dependent effects, and/or changes in environmental conditions have been proposed as possible factors underlying the recent reduction in Kemp’s ridley nest numbers [47]. Given relatively short reproductive longevity, delayed remigration could result in fewer nests overall during each female’s reproductive lifetime and a decrease in number of observed nests for the population.

### Growth dynamics

Comparable to results of previous Kemp’s ridley mark-recapture and skeletochronology studies, data yielded by the current analyses demonstrate that somatic growth is variable throughout life, as expected for ectotherms [13] and differs both among and within individuals, as observed in other sea turtle populations [22, 48]. The polyphasic growth pattern described by Chaloupka and Zug [49] is also indicated by the current data (Fig 2A), although the fluctuations in the size-at-age relationship are not as pronounced. GOM Kemp’s ridley somatic growth rates were found to be fastest for the smallest, youngest turtles, followed by a general decline (Fig 4A). However, transient increases in growth occurred, peaking between 25–30 cm SCL and around 45 cm SCL, and are potentially linked with ontogenetic habitat shifts and for the latter, changes associated with imminent onset of maturation [49]. Growth response for juvenile Kemp’s ridleys in the GOM appears to decrease overall starting around ages 4 and 5 (Fig 4B), consistent with previous findings for this species in the Atlantic [13].

Juvenile Kemp’s ridleys inhabit foraging areas both in the GOM and along the US Atlantic coast and questions have been raised regarding potential differences in somatic growth rates between the regions, as these have implications for differing ASMs and relative contributions to the reproductive population [13, 47, 50, 61]. Although some growth data have indicated that juvenile somatic growth rates in the GOM are higher, others suggest the converse is true [52]. While a full complement of data from all life stages is needed to accurately describe growth rates and predict ASM [52], sample availability and/or turtle distribution has constrained previous studies to specific size classes or life stages [13]. However, in the current study, it was possible to analyze samples for Kemp’s ridleys of all sizes, from hatchling to adult, with a very large sample size, to comprehensively characterize growth patterns in the GOM from 1988 to 2010. Comparison of juvenile Kemp’s ridley growth data available for the period prior to 2000 revealed that early growth in the GOM was faster than in the Atlantic, which could influence maturation trajectories and ASMs. However, additional data from Atlantic Kemp’s ridleys for the time period after 2000 and for the GOM after 2010 are required to provide an updated comparison.

The data presented herein indicate that within the GOM there have been long-term, negative trends in Kemp’s ridley somatic growth rates, with decreases after 2004–2005 for smaller juveniles (Fig 4C), and from 1988 to 2009 for large juveniles and adults (Fig 5). Although no GOM mark-recapture growth data spanning the same time frame are available to corroborate model predictions from the current study, Coleman et al. [53] recently reported growth rates for small, juvenile Kemp’s ridleys from 2010 to 2014 that are substantially lower compared to those observed previously in the GOM. The factors underlying decreased somatic growth for Kemp’s ridleys in the GOM are uncertain, yet there are a number of possibilities. Concurrent with extensive conservation efforts, from 1998 to 2010 the number of nests at the primary Kemp’s ridley nesting area increased exponentially, resulting in the release of hundreds of thousands of hatchlings each season [47]. Previous studies recognized that sufficient habitat and forage would be required to accommodate this influx of juveniles [54] and indicated that carrying capacity for the population might be reached at smaller sizes than in the past and that an abrupt “crash” might occur [10, 11]. Both juvenile and adult Kemp’s ridleys exhibit site fidelity to GOM foraging areas [54–60], with juveniles preferring shallower-water habitat [55, 57, 61, 62]. Furthermore, studies have reported that Kemp’s ridleys may specialize in their diet preferences, focusing on swimming crabs (in particular the blue crab, *Callinectes sapidus*), with additional consumption of various walking crab species [58, 63, 64]. This combination of fidelity to specific sites and forage species over long periods of time has the potential to limit flexibility in habitat shifts in response to environmental changes [59].

During the time frame encompassed by the current study (from 1988 to April 20, 2010, when the DWH oil spill occurred), a number of deleterious changes to environmental conditions in the GOM have been reported that could potentially reduce resources available to Kemp’s ridleys in the region. A large, seasonal hypoxic zone in the northwestern GOM began to increase in size during the 1980s and in recent years has been found to comprise an area as large as 22,000 km^2^ [65]. Hypoxia can displace benthic organisms to deeper, offshore waters [65] and/or concentrate organisms around the edges of the zone [66], reducing prey availability and increasing competition during foraging. During aerial surveys in the affected region, although turtles were observed to be abundant at 0 to 90 m depth and most common nearshore, none were observed in the hypoxic zone [66]. Furthermore, the GOM has seen an increase in hurricane activity since the 1990s and although storm events can sometimes be beneficial in terms of alleviating hypoxic or temperature stress [67], the passage of Hurricane Katrina in 2005 resulted in reductions in benthic community abundance, density, and diversity along the Louisiana and Mississippi coasts [68]. Overall decreases in GOM species richness, evenness, and diversity have been documented, particularly since 2002, with regional differences indicating location-specific influences of environmental changes and/or fishing effort [67]. In particular, both fishery-dependent and–independent data have indicated decreases in the blue crab population as far back as the 1980s [69–71], potentially resulting in limitations on availability of the preferred prey species for Kemp’s ridleys [72]. Also, Shaver [64] found evidence of consumption of shrimp trawl bycatch by Kemp’s ridleys stranded in Texas during the 1980s and 1990s. However, after 2000 when Turtle Excluder Device (TED) and Bycatch Reduction Device (BRD) use became mandatory for shrimp trawls, effort for this fishery in the Gulf of Mexico declined, reducing possible foraging opportunities for Kemp’s ridleys from this source.

Concurrent with this time frame, alterations in Kemp’s ridley habitat use and foraging behavior have also been observed. For example, in Texas, juveniles shifted from inhabiting tidal passes to increasing, yet divergent, use of both inshore bays and offshore areas, perhaps in response to variability in environmental conditions and prey quality or distribution [62]. Foraging studies have also indicated diversification of Kemp’s ridley diet, where in some areas the turtles are exhibiting preferences for walking crabs, molluscs, and even tunicates [54, 73]. Along the upper Texas and western Louisiana coasts and in west-central Florida, fish have been found to be prevalent in gastrointestinal tracts of stranded Kemp’s ridleys [58, 74]. In addition, large increases in bycatch of juvenile Kemp’s ridleys on recreational hook and line gear have occurred, potentially related to blue crab population fluctuations [75], with some turtles exhibiting site fidelity to areas around fishing piers, leading to repeated interactions with fishing gear [53]. As a result, these shifts to different habitats and forage items have the potential to result not only in changes in somatic growth rates, but may also expose the turtles to new threats to their survival [63].

## Conclusions

Results of the current study indicate substantial variability in Kemp’s ridley SSM and ASM, as well as long-term decreases in somatic growth rates, which have the potential to influence recruitment to the reproductive population. Recent tag return data from Texas indicate that the proportion of nesting females identified as neophytes has decreased over the last decade [46] and while a reduction in neophytes might be indicative of nesting population stability [76], lack of recruitment may contribute to observed fluctuations in nesting trends. Reproductive longevity was also variable and relatively short compared to larger sea turtle species, which has implications for lifetime reproductive output if resource limitations alter nesting frequency. Incorporation of this information into updated population models is needed to ascertain its potential influence on population dynamics. While this study mainly focused on the GOM prior to 2010, analysis of post-DWH oil spill GOM samples is needed to increase understanding of possible, subsequent changes in these population parameters. In addition, as samples from Texas and Florida predominated in the current study, more data from the northern GOM are needed to evaluate regional differences within this area. Furthermore, given that large numbers of juvenile Kemp’s ridleys are also present in foraging areas along the Atlantic US coast [51], there is a need to update age and growth data for juveniles in this area, to allow current comparison with the GOM, both prior and subsequent to the DWH oil spill. Finally, application of recent advances in integrating skeletochronology with stable isotope and trace element analyses [77] should be applied to increase understanding of long-term changes in trophic ecology for GOM Kemp’s ridleys, as well as movements between Atlantic and GOM foraging habitats and the relative contributions of these regions to the reproductive population [10].

### Appendix

As Lk ACC020422-02 was captured October 10, 1999 and as the majority of somatic growth for that season would have occurred prior to capture, the spring 2000 LAG was designated as that most representative of SCL at tagging. The 1999 LAG was unfortunately resorbed, not allowing growth rate back-calculation and therefore no adjustment was available for this turtle.

Lk DMD010708-01 was captured June 11, 2001 and therefore the spring 2001 LAG was most representative of tagging, corresponding with a back-calculated SCL of 29.3 cm. Based on the turtle’s SCL measurements at tagging and stranding July 8, 2001, it had grown 1.0 cm SCL in approximately 1 month. Assuming comparable growth between LAG deposition and tagging (~2 months), the adjusted SCL at tagging was estimated at 31.3 cm.

Lk BGS990404-01 was captured June 21, 1998 and therefore the spring 1998 LAG was most representative of tagging, corresponding with a back-calculated SCL of 30.6 cm. Between capture and stranding April 4, 1999 (9.5 mo), the turtle grew 9.4 cm, averaging approximately 1 cm SCL growth per month. Applying this growth rate between LAG deposition and tagging (~2 months), the adjusted SCL at tagging was estimated at 32.6 cm.

Lk AFA070427-03 was measured while nesting on Padre Island, TX, May 8, 2005 and therefore the spring 2005 LAG was most representative of this time. As this turtle’s back-calculated annual growth rate for 2005 was only 0.1 cm SCL, no adjustment was applied to the back-calculated SCL estimate.

LkAFA080516-01 was measured while nesting on South Padre Island, TX, June 23, 2007, and therefore the spring 2007 LAG was most representative of this time. Because this turtle’s back-calculated growth rate for 2007 was 0 cm SCL, no adjustment was applied to the back-calculated SCL estimate.

## Supporting information

S1 Fig Summary of Generalized Additive Mixed Model (GAMM) graphical output for models incorporating back-calculated somatic growth data for known-sex Gulf of Mexico Kemp’s ridley sea turtles (*Lepidochelys kempii*) <50 cm Straightline Carapace Length (notch-tip; SCL). Covariates include SCL, Age (yr), and calendar year (Year). Plots for those covariates exhibiting a significant influence on growth response are shown. Solid lines represent mean growth response centered around 0 and dashed lines represent the extent of the 95% Bayesian credible interval. The short, vertical lines above the horizontal axis (i.e., ‘rugs’) represent the distribution of samples for a given covariate. See S3 Table for sample sizes and statistical output.
(TIF)

S2 Fig Summary of Generalized Additive Mixed Model (GAMM) graphical output for models incorporating back-calculated somatic growth data for known-sex Gulf of Mexico Kemp’s ridley sea turtles (*Lepidochelys kempii*) >50 cm Straightline Carapace Length (notch-tip; SCL). Covariates include SCL or Age (yr), and calendar year (Year). Plots for those covariates exhibiting a significant influence on growth response are shown. Solid lines represent mean growth response centered around 0 and dashed lines represent the extent of the 95% Bayesian credible interval. The short, vertical lines above the horizontal axis (i.e., ‘rugs’) represent the distribution of samples for a given covariate. See S4 Table for sample sizes and statistical output.
(TIF)

S1 Table Results of validation analysis involving humeri collected from tagged, stranded Kemp’s ridley sea turtles (*Lepidochelys kempii*) and comparison of Straightline Carapace Length (SCL) measured at tagging with SCL back-calculated for the Line of Arrested Growth (LAG) deposited in the humerus closest to the time of tagging, as well as back-calculated SCL adjusted for growth between LAG deposition and tagging. (PDF)

S2 Table Summary of Generalized Additive Mixed Model (GAMM) statistical output for models incorporating back-calculated annual somatic growth data for all known-sex Gulf of Mexico Kemp’s ridley sea turtles (Lepidochelys kempii). Covariates include straightline carapace length (SCL) or Age, Sex, and calendar year (Year). Boxes enclose statistical output for the best-fitting models. AIC indicates Akaike’s information criterion values. For graphical summary, see Fig 4.
(PDF)

S3 Table Summary of Generalized Additive Mixed Model (GAMM) statistical output for models incorporating back-calculated somatic growth data for all known-sex Gulf of Mexico Kemp’s ridley sea turtles (Lepidochelys kempii) <50 cm Straightline Carapace Length (SCL). Covariates include SCL or Age, Sex, and calendar year (Year). Boxes enclose statistical output for the best-fitting models. AIC indicates Akaike’s information criterion values. For graphical summary, see S1 Fig.
(PDF)

S4 Table Summary of Generalized Additive Mixed Model (GAMM) statistical output for models incorporating back-calculated somatic growth data for all known-sex Gulf of Mexico Kemp’s ridley sea turtles (Lepidochelys kempii) >50 cm Straightline Carapace Length (SCL). Covariates include SCL or Age, Sex, and calendar year (Year). Boxes enclose statistical output for the best-fitting models. AIC indicates Akaike’s information criterion values. For graphical summary, see S2 Fig.
(PDF)
