# Supplementary figures and images for: Variability in age and size at maturation, reproductive longevity, and long-term growth dynamics for Kemp's ridley sea turtles in the Gulf of Mexico
Source: PLoS One. 2017 Mar 23;12(3):e0173999. doi: 10.1371/journal.pone.0173999 (PMC5363829; doi:10.1371/journal.pone.0173999)

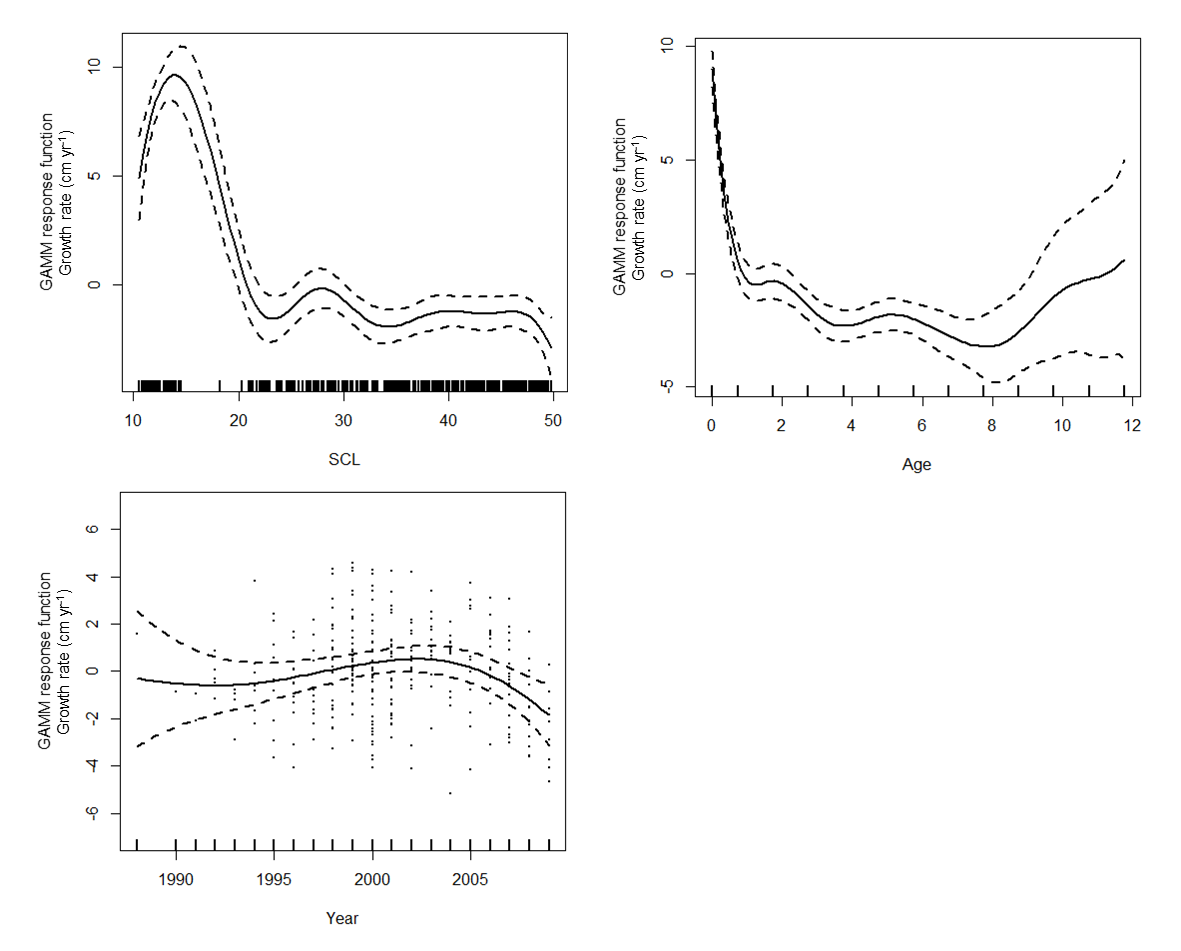

Supplement: S1 Fig — Covariates include SCL, Age (yr), and calendar year (Year). Plots for those covariates exhibiting a significant influence on growth response are shown. Solid lines represent mean growth response centered around 0 and dashed lines represent the extent of the 95% Bayesian credible interval. The short, vertical lines above the horizontal axis (i.e., ‘rugs’) represent the distribution of samples for a given covariate. See S3 Table for sample sizes and statistical output. (TIF) [file pone.0173999.s001.tif]

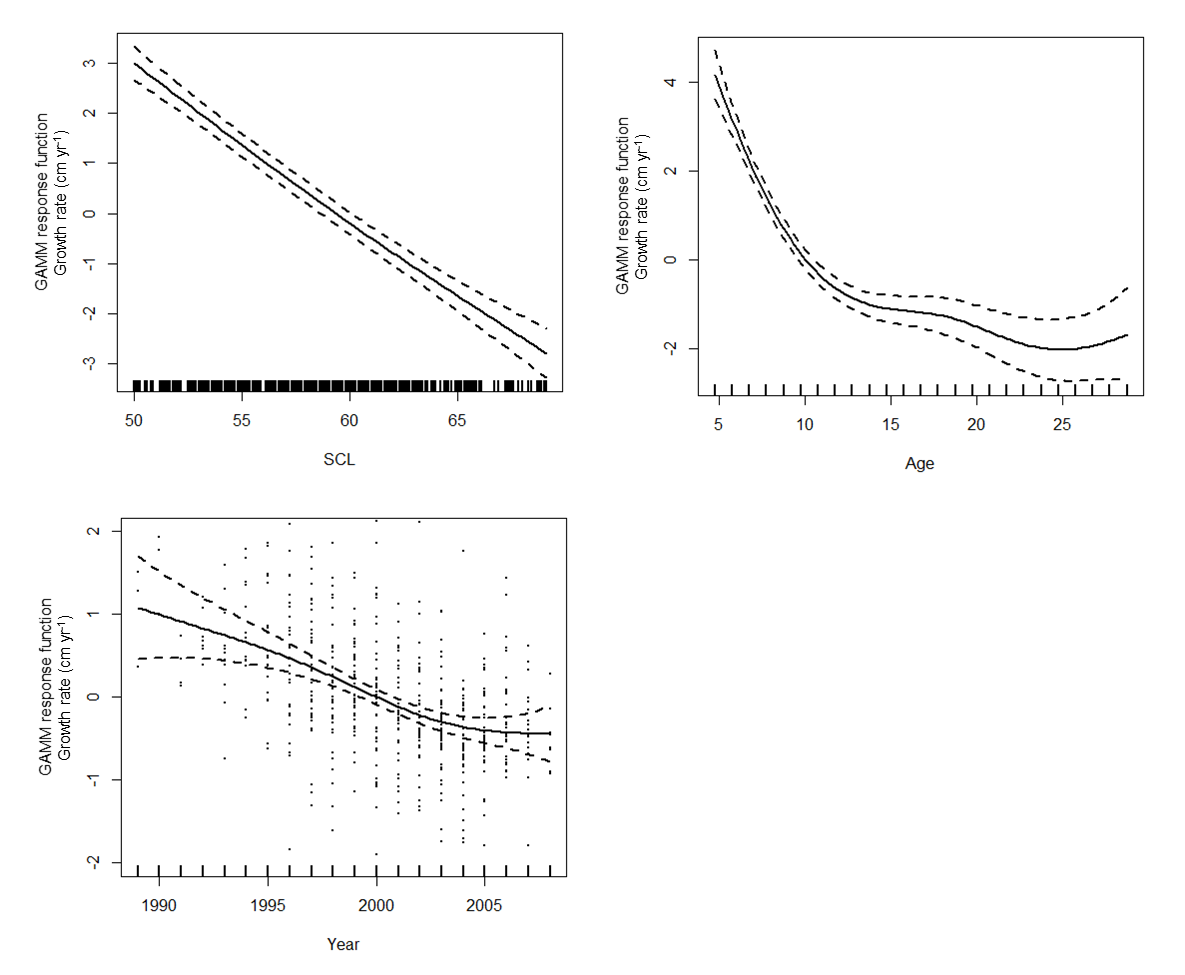

Supplement: S2 Fig — Covariates include SCL or Age (yr), and calendar year (Year). Plots for those covariates exhibiting a significant influence on growth response are shown. Solid lines represent mean growth response centered around 0 and dashed lines represent the extent of the 95% Bayesian credible interval. The short, vertical lines above the horizontal axis (i.e., ‘rugs’) represent the distribution of samples for a given covariate. See S4 Table for sample sizes and statistical output. (TIF) [file pone.0173999.s002.tif]
