# Supplementary material for: Variability in age and size at maturation, reproductive longevity, and long-term growth dynamics for Kemp's ridley sea turtles in the Gulf of Mexico
Source: PLoS One. 2017 Mar 23;12(3):e0173999. doi: 10.1371/journal.pone.0173999 (PMC5363829; doi:10.1371/journal.pone.0173999)
Supplement: S1 Table — (PDF) [file pone.0173999.s003.pdf]

Turtle ID	Capture date	Measured SCL (cm)	Estimated SCL (cm)	Back-calculated growth rate (cm/month)	Estimated growth between LAG deposition and tagging (cm)	Estimated + adjusted (cm)	Difference: Measured - Estimated (cm)	Difference: Measured - Estimated + adjusted (cm)
Lk ACC020422-02	10/10/1999	31.2	31.9	*	*	31.9	0.7	0.7
Lk DMD010708-01	6/11/2001	31.2	29.3	1	2	31.3	1.9	0.1
Lk BG990404-01	6/21/1998	32.5	30.6	1	2	32.6	1.9	0.1
Lk AFA070427-03	5/8/2005	64.2	65.2	0	0	65.2	1	1
Lk AFA080516-01	6/23/2007	68.7	69	0	0	69	0.3	0.3
							Paired t-test *p = 0.60*	*Wilcoxon Signed Rank Test* *p = 1.00*

*Adjustment not available; prior growth rate could not be calculated because LAG resorbed
