# Supplementary material for: Variability in age and size at maturation, reproductive longevity, and long-term growth dynamics for Kemp's ridley sea turtles in the Gulf of Mexico
Source: PLoS One. 2017 Mar 23;12(3):e0173999. doi: 10.1371/journal.pone.0173999 (PMC5363829; doi:10.1371/journal.pone.0173999)
Supplement: S2 Table — Covariates include straightline carapace length (SCL) or Age, Sex, and calendar year (Year). Boxes enclose statistical output for the best-fitting models. AIC indicates Akaike’s information criterion values. For graphical summary, see Fig 4. (PDF) [file pone.0173999.s004.pdf]

Model	n	Adjusted r^2	AIC	*Smooth terms* Variable	Edf	*F*	Prob(*F*)	*Parametric coefficients* Variable	Estimate	Std Error	*t*	Pr>|*t*|
Sex + SCL + Year	828	0.76	3289	*SCL*	8.917	228.585	<0.001	*Constant*	4.026	0.191	12.053	<0.001
				Year	1.001	0.968	0.33	*Sex_male*	-0.272	0.273	-0.996	0.32
SCL + Year	828	0.76	3288	*SCL*	8.916	229.374	<0.001	*Constant*	3.898	0.141	27.57	<0.001
				Year	1.001	0.842	0.36					
SCL	828	0.766	3285	*SCL*	8.915	254.1	<0.001	*Constant*	3.909	0.138	28.39	<0.001
Year	828	-0.187	4173	*Year*	7.201	23.44	<0.001	*Constant*	5.703	0.378	15.07	<0.001
Sex + Age + Year	828	0.796	3444	*Age*	8.652	347.134	<0.001	*Constant*	3.495	0.09	38.829	<0.001
				Year	5.783	7.354	<0.001	*Sex_male*	0.023	0.132	0.176	0.86
Age + Year	828	0.796	3442	*Age*	8.653	347.971	<0.001	*Constant*	3.507	0.064	55.03	<0.001
				Year	5.783	5.576	<0.001					
Age	828	0.787	3458	*Age*	8.673	334.6	<0.001	*Constant*	3.526	0.07	50.39	<0.001
Year	828	-0.187	4173	*Year*	7.201	15.07	<0.001	*Constant*	5.703	0.378	15.07	<0.001
