# Supplementary material for: Variability in age and size at maturation, reproductive longevity, and long-term growth dynamics for Kemp's ridley sea turtles in the Gulf of Mexico
Source: PLoS One. 2017 Mar 23;12(3):e0173999. doi: 10.1371/journal.pone.0173999 (PMC5363829; doi:10.1371/journal.pone.0173999)
Supplement: S3 Table — Covariates include SCL or Age, Sex, and calendar year (Year). Boxes enclose statistical output for the best-fitting models. AIC indicates Akaike’s information criterion values. For graphical summary, see S1 Fig. (PDF) [file pone.0173999.s005.pdf]

GAMM <50 cm SCL

Model	n	Adjusted r^2	*Smooth terms*					*Parametric coefficients*				
			AIC	Variable	Edf	*F*	Prob(*F*)	Variable	Estimate	Std Error	*t*	Pr>|*t*|
Sex + SCL + Year	303	0.656	1471	SCL	7.636	81.187	<0.001	Constant	7.461	0.275	27.167	<0.001
				Year	3.274	2.877	0.03	Sex_male	0.061	0.413	0.147	0.88
SCL + Year	303	0.657	1469	SCL	7.637	79.891	<0.001	Constant	7.488	0.202	36.99	<0.001
				Year	3.279	2.832	0.03					
SCL	303	0.65	1469	SCL	7.62	79.31	<0.001	Constant	7.465	0.204	36.63	<0.001
Year	303	0.046	1759	Year	5.619	5.05	<0.001	Constant	7.618	0.334	22.78	<0.001
Sex + Age + Year	303	0.702	1460	Age	8.023	82.88	<0.001	Constant	7.295	0.246	29.226	<0.001
				Year	3.143	3.9	0.008	Sex_male	0.314	0.37	0.848	0.4
Age + Year	303	0.702	1458	Age	8.02	83.04	<0.001	Constant	7.337	0.181	40.44	<0.001
				Year	3.169	3.84	0.009					
Age	303	0.689	1461	Age	8.012	81.16	<0.001	Constant	7.342	0.187	39.19	<0.001
Year	303	0.046	1759	Year	5.619	5.05	<0.001	Constant	7.618	0.334	22.78	<0.001
