# Supplementary material for: Variability in age and size at maturation, reproductive longevity, and long-term growth dynamics for Kemp's ridley sea turtles in the Gulf of Mexico
Source: PLoS One. 2017 Mar 23;12(3):e0173999. doi: 10.1371/journal.pone.0173999 (PMC5363829; doi:10.1371/journal.pone.0173999)
Supplement: S4 Table — Covariates include SCL or Age, Sex, and calendar year (Year). Boxes enclose statistical output for the best-fitting models. AIC indicates Akaike’s information criterion values. For graphical summary, see S2 Fig. (PDF) [file pone.0173999.s006.pdf]

Turtle ID	Capture date	Measured SCL (cm)	Estimated SCL (cm)	Back-calculated growth rate (cm/month)	Estimated growth between LAG deposition and tagging (cm)	Estimated + adjusted (cm)	Difference: Measured - Estimated (cm)	Difference: Measured - Estimated + adjusted (cm)
Lk ACC020422-02	10/10/1999	31.2	31.9	*	*	31.9	0.7	0.7
Lk DMD010708-01	6/11/2001	31.2	29.3	1	2	31.3	1.9	0.1
Lk BG990404-01	6/21/1998	32.5	30.6	1	2	32.6	1.9	0.1
Lk AFA070427-03	5/8/2005	64.2	65.2	0	0	65.2	1	1
Lk AFA080516-01	6/23/2007	68.7	69	0	0	69	0.3	0.3
							Paired t-test *p = 0.60*	*Wilcoxon Signed Rank Test* *p = 1.00*

*Adjustment not available; prior growth rate could not be calculated because LAG resorbed

Model	n	Adjusted r^2	AIC	*Smooth terms* Variable	Edf	*F*	Prob(*F*)	*Parametric coefficients* Variable	Estimate	Std Error	*t*	Pr>|*t*|
Sex + SCL + Year	828	0.76	3289	*SCL*	8.917	228.585	<0.001	*Constant*	4.026	0.191	12.053	<0.001
				Year	1.001	0.968	0.33	*Sex_male*	-0.272	0.273	-0.996	0.32
SCL + Year	828	0.76	3288	*SCL*	8.916	229.374	<0.001	*Constant*	3.898	0.141	27.57	<0.001
				Year	1.001	0.842	0.36					
SCL	828	0.766	3285	*SCL*	8.915	254.1	<0.001	*Constant*	3.909	0.138	28.39	<0.001
Year	828	-0.187	4173	*Year*	7.201	23.44	<0.001	*Constant*	5.703	0.378	15.07	<0.001
Sex + Age + Year	828	0.796	3444	*Age*	8.652	347.134	<0.001	*Constant*	3.495	0.09	38.829	<0.001
				Year	5.783	7.354	<0.001	*Sex_male*	0.023	0.132	0.176	0.86
Age + Year	828	0.796	3442	*Age*	8.653	347.971	<0.001	*Constant*	3.507	0.064	55.03	<0.001
				Year	5.783	5.576	<0.001					
Age	828	0.787	3458	*Age*	8.673	334.6	<0.001	*Constant*	3.526	0.07	50.39	<0.001
Year	828	-0.187	4173	*Year*	7.201	15.07	<0.001	*Constant*	5.703	0.378	15.07	<0.001

GAMM <50 cm SCL

Model	n	Adjusted r^2	Smooth terms					Parametric coefficients				
			AIC	Variable	Edf	*F*	Prob(*F*)	Variable	Estimate	Std Error	*t*	Pr>|*t*|
Sex + SCL + Year	303	0.656	1471	SCL	7.636	81.187	<0.001	Constant	7.461	0.275	27.167	<0.001
				Year	3.274	2.877	0.03	Sex_male	0.061	0.413	0.147	0.88
SCL + Year	303	0.657	1469	SCL	7.637	79.891	<0.001	Constant	7.488	0.202	36.99	<0.001
				Year	3.279	2.832	0.03					
SCL	303	0.65	1469	SCL	7.62	79.31	<0.001	Constant	7.465	0.204	36.63	<0.001
Year	303	0.046	1759	Year	5.619	5.05	<0.001	Constant	7.618	0.334	22.78	<0.001
Sex + Age + Year	303	0.702	1460	Age	8.023	82.88	<0.001	Constant	7.295	0.246	29.226	<0.001
				Year	3.143	3.9	0.008	Sex_male	0.314	0.37	0.848	0.4
Age + Year	303	0.702	1458	Age	8.02	83.04	<0.001	Constant	7.337	0.181	40.44	<0.001
				Year	3.169	3.84	0.009					
Age	303	0.689	1461	Age	8.012	81.16	<0.001	Constant	7.342	0.187	39.19	<0.001
Year	303	0.046	1759	Year	5.619	5.05	<0.001	Constant	7.618	0.334	22.78	<0.001

**GAMM >50 cm SCL**				*Smooth terms*				*Parametric coefficients*				
Model	n	Adjusted r^2	AIC	Variable	Edf	*F*	Prob(*F*)	Variable	Estimate	Std Error	*t*	Pr>|*t*|
Sex + SCL + Year	525	0.45	1456	*SCL*	1.679	320.710	<0.001	*Constant*	1.511	0.150	10.105	<0.001
				Year	2.692	11.740	<0.001	*Sex_male*	-0.056	0.210	-0.268	0.79
SCL + Year	525	0.449	1454	*SCL*	1.715	317.420	<0.001	*Constant*	1.483	0.106	14.050	<0.001
				Year	2.698	12.070	<0.001					
SCL	525	0.327	1476	*SCL*	1.000	960.500	<0.001	*Constant*	1.379	0.118	11.650	<0.001
Year	525	-0.333	1777	*Year*	2.777	134.400	<0.001	*Constant*	2.066	0.191	10.800	<0.001
Sex + Age + Year	525	0.524	1510	*Age*	5.278	81.350	<0.001	*Constant*	1.528	0.135	11.313	<0.001
				Year	3.268	7.070	<0.001	*Sex_male*	-0.050	0.191	-0.264	0.79
Age + Year	525	0.524	1508	*Age*	5.278	82.842	<0.001	*Constant*	1.503	0.096	15.680	<0.001
				Year	3.267	7.132	<0.001					
Age	525	0.499	1522	*Age*	5.113	154.500	<0.001	*Constant*	1.445	0.095	15.230	<0.001
Year	525	-0.333	1777	*Year*	2.777	134.400	<0.001	*Constant*	2.066	0.191	10.800	<0.001
